# The microRNA-205-5p is correlated to metastatic potential of 21T series: A breast cancer progression model

**DOI:** 10.1371/journal.pone.0173756

**Published:** 2017-03-27

**Authors:** L. Stankevicins, A. Barat, P. Dessen, Y. Vassetzky, C. V. de Moura Gallo

**Affiliations:** 1 Departamento de Genética, Universidade do Estado do Rio de Janeiro, Instituto de Biologia Roberto Alcantara Gomes, Rio de Janeiro, Brazil; 2 CNRS UMR 8126 «Signalisation, noyaux et innovations en cancérologie», Université Paris-Sud, Institut de Cancérologie Gustave-Roussy, Villejuif cedex, France; 3 Functional Genomics Unit, Institut de Cancérologie Gustave-Roussy, Villejuif, France; 4 N.K. Koltzov Institute of Developmental Biology RAS, Moscow, Russia; King Saud University, SAUDI ARABIA

## Abstract

MicroRNA is a class of noncoding RNAs able to base pair with complementary messenger RNA sequences, inhibiting their expression. These regulatory molecules play important roles in key cellular processes including cell proliferation, differentiation and response to DNA damage; changes in miRNA expression are a common feature of human cancers. To gain insights into the mechanisms involved in breast cancer progression we conducted a microRNA global expression analysis on a 21T series of cell lines obtained from the same patient during different stages of breast cancer progression. These stages are represented by cell lines derived from normal epithelial (H16N2), atypical ductal hyperplasia (21PT), primary *in situ* ductal carcinoma (21NT) and pleural effusion of a lung metastasis (21MT-1 and 21MT-2). In a global microRNA expression analysis, miR-205-5p was the only miRNA to display an important downregulation in the metastatic cell lines (21MT-1; 21MT-2) when compared to the non-invasive cells (21PT and 21NT). The lower amounts of miR-205-5p found also correlated with high histological grades biopsies and with higher invasion rates in a Boyden chamber assay. This work pinpoints miR-205-5p as a potential player in breast tumor invasiveness.

## Introduction

Breast cancer is the most frequent carcinoma in women and a highly heterogeneous disease. Diagnostic and treatment decision are mainly based on classical biological variables including morphology, tumor grade, presence of lymph-node metastasis and molecular markers [1]. Cancer progression is a multistep process, progressing from normal epithelia to an atypical ductal hyperplasia then to ductal carcinoma *in situ* (DCIS), invasive ductal carcinoma (IDC) culminating in metastasis [2]. The knowledge on the DCIS transition to IDC is still incomplete and there are many questions concerning breast cancer progression. It is not clear if the enrichment of few specific genes in IDC is enough to promote cell migration or if other factors such as the microenvironment, breast stem cells enrichment or epigenetic changes, including microRNA (miRNA) regulation, are acting together to promote metastasis.

miRNAs are small (about 23 nucleotides long) non-coding RNA species that have emerged as major elements of gene expression control, acting in post-transcriptional level by targeting complementary mRNA sequence [3]. miRNAs are often altered in breast cancer and can have either tumor suppressor or oncogenic activity being able to modulate nearly all relevant stages of cancer progression including cell proliferation, apoptosis, cell migration, angiogenesis and stem cell maintenance [3, 4]. In breast tumors, miR-200 family, miR-21 and miR-205-5p were shown to regulate cell proliferation and invasion [5, 6]. The miR-200 family and miR-205-5p can modulate epithelial to mesenchymal transition (EMT) mainly by e-cadherin regulation via ZEB-1 inhibition [5]. Also, miR-205-5p can be negatively regulated by HER-2, possibly contributing for the worse prognostic associated to HER-2 enriched subtype [7]. To identify differentially expressed miRNAs in breast cancer progression, we used as experimental model the 21T series of cell lines which is an *in vitro* model of breast cancer progression comprising cell lines derived from the same patient which include a normal epithelia (H16N2), atypical ductal hyperplasia (21PT), primary *in situ* ductal carcinoma (21NT) and cells derived from pleural effusion of lung metastasis (21MT-1 and 21MT-2) [8]. This is an excellent *in vitro* model to describe *de novo* processes occurring during breast cancer evolution, excluding the influence of different genetic backgrounds [9].

The present study was aimed to evaluate changes in microRNA expression in the 21T series of cell lines, representing different stages of breast cancer progression, and accumulate evidence of a key role of miR-205-5p in cell migration and metastasis.

## Material and methods

### Cell lines and culture

The 21T series of cell lines were kindly provided from Dr. Pierre Hainaut (IARC-Lyon, France) and Dr. Vimla Band (University of Nebraska Medical Center, USA). Cells were cultured in DMEM-F12 media (Thermo Fisher Scientific, Waltham, MA, USA) supplemented with 10% FBS, EGF (20ng/mL), insulin (10mg/mL) and hydrocortisone (0.5mg/mL) (Sigma-Aldrich, St Louis, MO, USA) at 37^°^C and 5% CO_2_. All cell cultures were routinely checked for mycoplasma contamination.

### Tissue samples

Paraffin embedded formalin fixed biopsies from normal breast epithelia (6 samples) and breast cancer cases diagnosed as infiltrating ductal carcinoma (10 samples) were obtained from the Department of Pathology, Fernandes Figueira Institute (IFF-FIOCRUZ), in Rio de Janeiro. The patient’s data were obtained from the hospital records. This study was approved by the Ethics Committee of the Fernandes Figueira Institute- IFF, FIOCRUZ-Rio de Janeiro and all participants signed an informed consent.

### RNA extraction

The extraction of total RNA from cell cultures was performed with Trizol reagent (Life Technologies). The amount and quality of RNA samples were assessed using the Agilent 2100 Bioanalyzer with RNA 6000 Nano Reagents and Supplies (Agilent Technologies, Santa Clara, CA, USA). Total RNA was isolated from formalin fixed paraffin embedded samples using High Pure RNA Paraffin Kit (Roche Applied Science, Penzberg, Germany).

### miRNA microarray

The miRNA global expression analysis was performed using the SurePrint Human miRNA Microarrays assays- Human miRNA Microarray Release 16.0, 8x60K. Based on miRBase version 16.0. (Agilent Technologies, Santa Clara, CA, USA).

Briefly, total RNA samples (100ng each) were dephosphorylated and labeled with the fluorophore Cyanine 3- pCp at the 3'end of the RNA molecule. After labeling, samples were purified with Micro Bio-spin 6 columns (Bio-Rad) and hybridized on array slides at 55^°^C, 20 rpm for 20h. The label and hybridization reactions were performed using miRNA Complete Labeling and Hyb Kit (Agilent Technologies, Santa Clara, CA, USA). The microarray slides were scanned on Agilent microarray scanner version C.

### Microarray data analysis

To perform microarray data analysis we used R language and the Limma package on Bioconductor software essentially as described in [10]. Data were background corrected using AgiMicroRNA algorithm and normalized between arrays by the quantile method in order to compensate for systematic technical differences between probe affinity and arrays. After background correction, differential expression was analyzed using the Limma package. A 2.0 times fold expression cutoff was applied to minimize the effect of probe background signal.

### Quantitative RT-PCR

For miRNAs quantification, cDNA synthesis was carried out with total RNA obtained from the 21T cell lines and tissue biopsies, using NCode VILO miRNA cDNA Synthesis Kit (Thermo Fisher Scientific, Waltham, MA, USA). For mRNAs quantification, cDNA synthesis was achieved, using High Capacity cDNA Reverse Transcription Kit (Thermo Fisher Scientific, Waltham, MA, USA). Real-time PCR was performed using SYBR PCR Master Mix (Thermo Fisher Scientific, Waltham, MA, USA). The primers sequences employed in qRT-PCR reactions were: b-actin (F) 5´ CATCGAGCACGGCATCGT 3´; b-actin (R) 5´ GCCTGGATAGCAACGTACAT 3´, miR-205-5p (F) 5´ CTTCATTCCACCGGAGTCTG 3´ with the universal reverse primer provided on miRNA cDNA synthesis kit. All experiments were performed in triplicate. β- actin was chosen as reference gene since its expression levels were constant in our experimental conditions. Expression results were analyzed using the ΔΔCt method [11] and statistical analysis was done using unpaired T-test.

### Invasion assay

For cell invasion assay, 0.5 x 10^4^ cells were seeded on the upper container of 8μm pore Boyden chamber (Merck Millipore, Darmstadt, Germany) coated with 10% Matrigel (BD Biosciences Franklin Lakes, NJ, USA), diluted in the respective culture media. Cells were incubated in a starvation media, without fetal calf serum for 22h at 37^°^C, 5% CO_2_ and the recipient dish underneath the cells was filled with a nutrient media containing 10% fetal calf serum. The non-migrating cells were removed from the upper part of the chamber with a cotton swab. Cells able to transmigrate to the lower part of the chamber were counted after being fixed with 4% paraformaldehyde and having the nucleus stained by Hoechst 3342. Results are displayed as a percentage ratio between tumoral and the normal cell line H16N2 and between the respective non-treated control in the functional assays.

### miR-205-5p target prediction

To increase the accuracy in miR-205-5p *in silico* target prediction we used as approach a combined miRNA and mRNA transcriptome profiling [10]. First, the mRNAs that had the expression increased more than two fold in 21T cells during the transition *in situ* to metastatic tumor (21MT1 vs 21NT) were selected from a published database [8]. After this first selection, we performed a search for miR-205-5p binding sites among the overexpressed mRNA, using the software RNA22 [12]. The most likely interaction sites (p≤0.05) were considered as possible miR-205-5p target.

## Results

### Differential expression of miRNAs in 21T series reveals miR-205-5p downregulation during breast cancer progression

**Fig 1:**
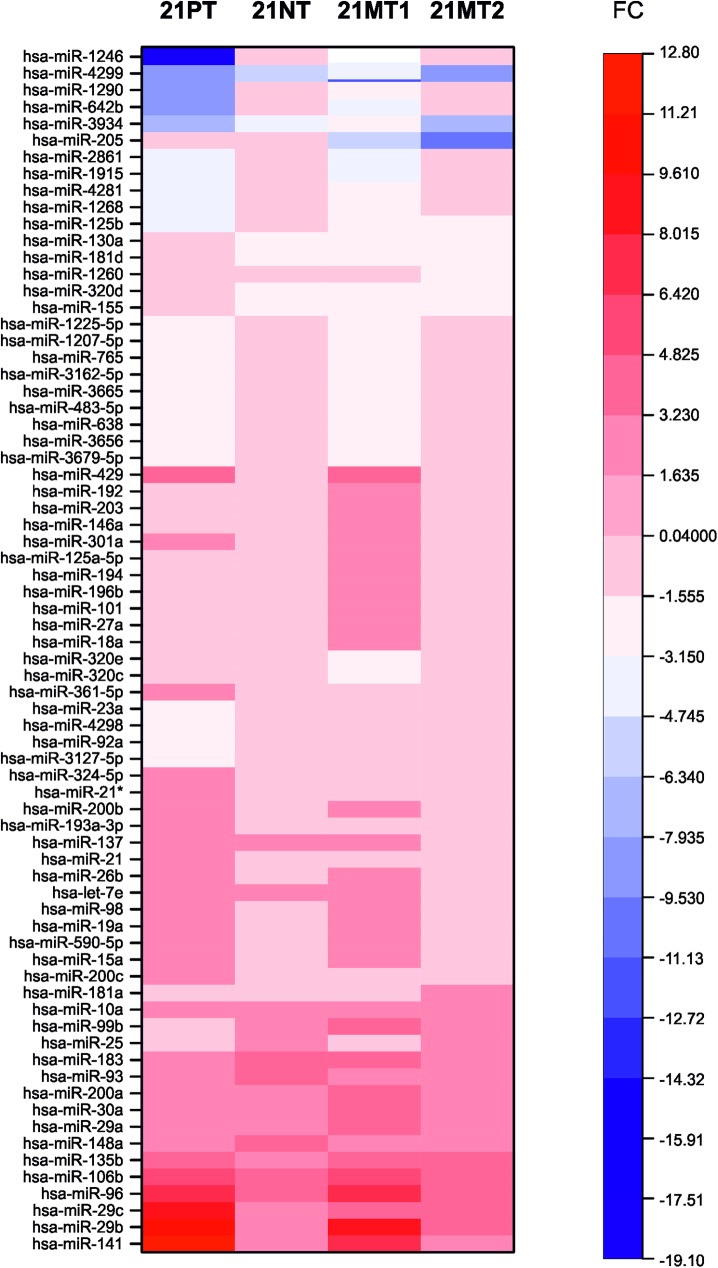
miRNA profiling in different stages of breast tumor progression. miRNA expression levels were assessed by microarray. The heatmap display all the miRNAs that presented altered expression levels (p≤0.05; cut-off 2.0-fold change) when compared to the normal epithelial cell line (H16N2).

We assessed the global miRNA expression during breast cancer progression in 21T cells using miRNA Human miRNA Microarray Release 16.0 microarray assay (Agilent Technologies, Santa Clara, CA, USA). Microarray data are available in the ArrayExpress database (http://www.ebi.ac.uk/arrayexpress) under accession number E-MTAB-5239. The comparison between all cell lines representing the different stages of tumor progression (21PT, 21NT, 21MT1 and 21MT2) and the cell line obtained from the normal breast tissue (H16N2) revealed a large number of deregulated miRNAs (Fig 1, Figs A-D in S1 File).

The transition from normal tissue to hyperplasia (H16N2-21PT) caused a significant increase in the expression levels (≥ 2 times fold up-regulated) of 29 miRNAs (Fig 1, Fig A in S1 File). The miR-141, miR-29 family, miR-96 and miR-106b presented the highest increase in the expression levels (≥ 5 times fold up-regulated). Those early stage up-regulation persisted at all stages of tumor progression (Fig 1, Figs A-D in S1 File).

Among the down-regulated miRNAs, the miR-4299 (≤ 6 times fold down-regulated) and miR-3934 (≤ 2.6 down-regulated) were altered throughout all stages of tumor progression (Fig 1, Figs A-D in S1 File).

**Fig 2:**
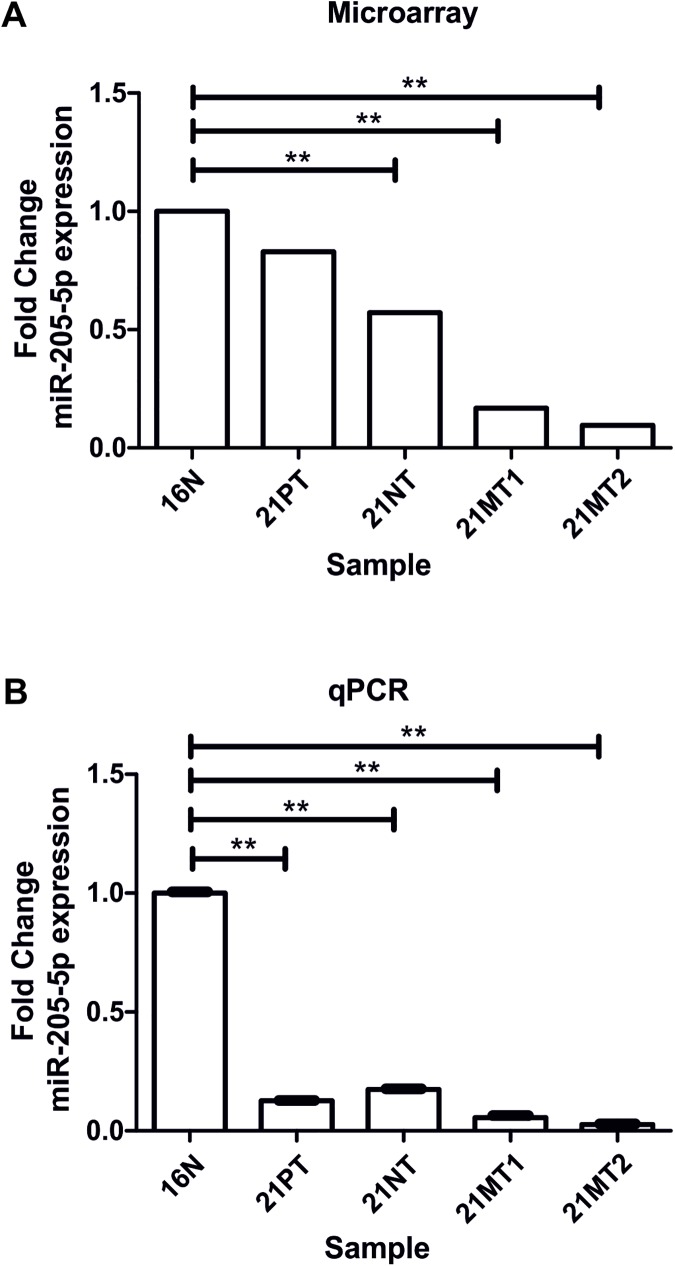
Downregulation of miR-205-5p during breast cancer progression in 21T series. miRNA expression levels in 21Tcells, relative to the non-tumorigenic cell line H16N2 quantified by **(A)** microarray and **(B)** qPCR. ** t- test p ≤ 0.01. Microarray experiment was done in duplicate and expression levels of miR-205-5p was confirmed by qPCR in 3 independent measures. Microarray analysis (p≤0.05; cut-off 2.0-fold change) revealed miR-205-5p as the only down-regulated miRNA in the comparison between 21PT and 21NT *in situ vs*. 21MT1 and 21MT2 metastatic showing 5.42 fold change p- value 0.003.

miR-205-5p presented a progressive downregulation during tumor progression (Fig 2A). The comparison between *in situ* tumoral cells (21PT/21NT) with metastatic cells (21MT1/21MT2) surprisingly revealed miR-205-5p as the only differentially expressed miRNA, with a 5.4 times downregulation (Fig 2A).

The validation of microarray data was done by qPCR confirming that miR-205-5p is down-regulated through breast cancer progression to metastasis (Fig 2B).

### miR-205-5p downregulation is observed in high-grade breast cancer biopsies

**Fig 3:**
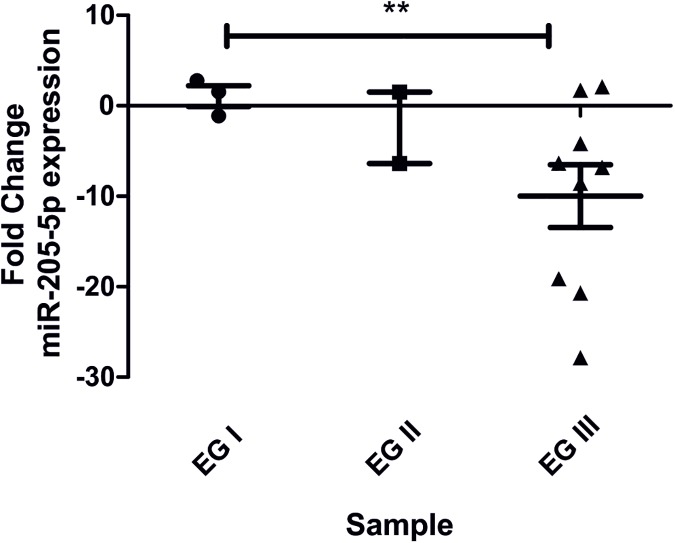
MiR-205-5p expression levels in paraffin-embedded formalin fixed biopsies in breast cancer cases of invasive ductal carcinomas, classified according to Elston grade (EG). The EG histological classification takes into account the tissue differentiation, nuclear pleomorphism and the mitotic rate. Tumors classified, as EG III are more proliferative and aggressive. miR-205-5p expression levels were obtained by qRT-PCR and the expression fold change was calculated in comparison to the average of miR-205-5p expression in non-tumoral breast biopsies. ** t- test p ≤ 0.01.

miR-205-5p expression can be related to tumor aggressiveness *in vivo*. miR-205-5p expression was analyzed by qRT-PCR in paraffin embedded formalin fixed biopsies of breast tissue samples (S5 Table with patient description and tumor characteristics). Breast cancer cases diagnosed as invasive ductal carcinoma were distributed according Elston’s classification in I, II and III [13]. Elston grade III representing the most proliferative and aggressive stage. miR-205-5p fold change in breast cancer tissue samples were calculated in comparison to miR-205-5p mean expression of 6 biopsies obtained from normal breast tissues (Fig A in S2 File) for the non- normalized distribution of the miR-205-5p expression). As shown in Fig 3, we could demonstrate a significant downregulation of miR-205-5p displayed in the most aggressive Elston grade III breast tumors in comparison to normal breast tissues.

### miR-205-5p low expression correlates to 21T cells invasive phenotype

**Fig 4:**
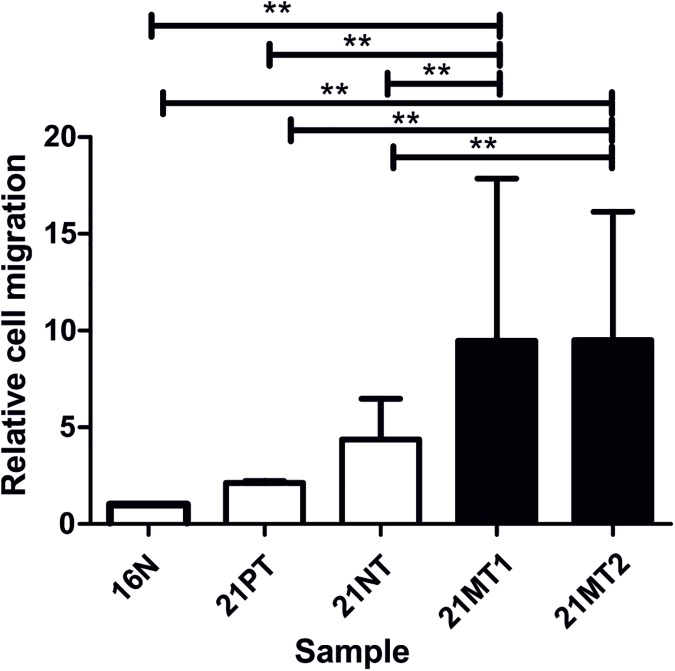
21T series show increased invasive patterns from non-tumoral to metastatic cell lines. 21T cells migrated through an 8.0μm pore size polycarbonate membrane coated with Matrigel. The number of migrating cells were assessed after 22h incubation at 37^°^C, 5% CO_2._ Comparisons where the changes in the migration rate were highly significant (t-test; p ≤ 0.01) are represented by connecting lines. Results are displayed as a percentage ratio between tumoral and the normal cell line H16N2. Error bars display standard deviation of the ratio. The mean values (M) and coefficient of variation (CV) of invasiveness in the 21T cell lines were: H16N2- M: 4.3 cells CV: 0.81; 21PT- M: 9.5 cells CV: 0.46; 21NT- M: 3.9 cells CV: 0.15; 21MT1- M: 66.7 cells CV: 0.17; 21MT2- M:18.0 cells CV: 0.3.

The 21T invasive phenotype was measured by the capacity of the 21T cells to transmigrate through 8μm pored membrane coated with 10% Matrigel matrix. The cells were seeded on top of an 8μm pored membrane coated with 10% Matrigel matrix. The percentage of 21T cells able to transmigrate to the lower part of the membrane is shown in Fig 4. These results confirm our assumption that miR-205-5p expression levels are inversely related to the invasion capacity. To verify if the exclusive change in miR-205-5p expression levels is able to alter the 21T cells invasiveness, we transfected 21MT1 and 21MT2 cells with the miR-205-5p precursor and H16N2, 21PT and 21NT cells with a miR-205-5p silencer. miR-205-5p silencing in the non- invasive cell lines (H16N2, 21PT and 21NT) partially increased migration rates while miR-205-5p overexpression in metastatic cells (21MT1 and 21MT2) reduced their migration capacity (S1 Fig). Although not statistically significant, these observations suggest that miR-205-5p plays an important role reverting breast cancer cells to a less aggressive behavior.

### miR-205-5p has potential cancer-related targets

A simultaneous correlation between mRNA overexpression and miR-205-5p downregulation during *in situ* to metastatic transition in 21T cells was used as a filter for a subsequent identification of miR-205-5p binding sites in the selected mRNAs. Eight miR-205-5p potential target mRNAs were selected (Table 1). A total of eleven miR-205-5p binding sites were identified using this approach (S1 Table). The predicted miR-205-5p targets comprises genes with functions related to cell invasion such as SOCS3, PTPRN2, and MMP3 and on epithelial to mesenchymal transition such as TGFB1 (Table 1).

**Table 1:** Predicted miR-205-5p genes in 21T cell lines. List of genes containing miR-205-5p binding sites. ^1^ Number of miR-205-5p binding sites predicted by RNA22 software. ^2^ mRNA fold change in 21MT1 when compared to 21NT, data obtained from [8].

Gene name (symbol)	Cancer-related gene ontology	Number of miR-205-5p predicted binding sites^1^	mRNA Fold Change (21MT1 *vs*. 21NT)^2^
SOCS3	Cell growth and invasion	3	7.01
TGFB1	EMT, Cell invasion	2	3.65
PTPRN2	Cell invasion and apoptosis resistance	1	20.15
MMP3	Cell invasion	1	7.41
PRG1	Tumor suppressor gene	1	5.89
BASP1	Tumor suppressor gene	1	4.23
ENO2	not described	1	10.08
TOSO	not described	1	5.07

## Discussion

Breast cancer is one of the most common cancer type concerning women worldwide [14]. Tumor progression for a metastatic phenotype is the major complication causing patient death. The high heterogeneity among breast tumors is a challenge for the development of more specific therapies and for the identification of consistent prognostic biomarkers. In this sense, we conducted a study to detect differences in microRNA expression during breast cancer progression using the 21T series of cell lines [15, 16]. The 21T cells were isolated from the same patient at different tumor stages. This allowed us to map the changes in miRNAs expression during the tumor progression, excluding inter-individual genetic differences. The 21T series is composed of five cell lines: H16N2, representative of adjacent non-tumoral breast cells; 21PT, representative of Atypical Ductal Hyperplasia (ADH) cells; 21NT of Ductal Carcinoma *in Situ* (DCIS) and 21MT1 and 21MT2, of Invasive Metastatic Carcinoma (IMC) [8]. Previous research have demonstrated that this series of cell lines is a very interesting model to investigate breast cancer progression [8, 16] and epigenetic alterations [9]. We have recently demonstrated a global hypomethylation pattern as well as chromatin changes, such as the accumulation of pericentric heterochromatin in metastatic 21MT1 cells [9].

We have first analyzed miRNA expression patterns in the five cell lines using microarray expression assay.

Among the altered miRNAs, miR-4299 and miR-3934 presented a strong downregulation in all tested cell lines, during all stages of tumor progression. *In silico* target prediction combined with gene ontology analysis reveals that miR-4299 can potentially regulate ErbB signaling pathway in different points by targeting BRAF, EIF4EBP1, PIK3R3 and BTC [17]. miR-3934 predicted targets include components of the oncogenic Wnt signaling pathway such as WNT7, WNT9A, MAPK10, APC and ROCK2 [17]. The up-regulated miRNAs in all 21T cell lines, suggesting an early event in tumorigenesis include the oncomiRs miR-29a/b/c [18, 19], miR-141 [20, 21], miR106b [22] and miR-96 [23, 24]. In this study, we focus our attention at the miRNA profile during the progression from an *in situ* tumor to a metastatic phenotype. The downregulation of miR-205-5p observed between 21PT/21NT and 21MT1/21MT2 cells, representing the transition hyperplasia/*in situ* to metastasis suggest a specific role of miR-205-5p in cell invasion. miR-205-5p down-regulation was later confirmed by qRT-PCR (Fig 2C). Indeed, miR-205-5p expression decreased with the tumor stage from Elston histological grade I through III (Fig 3), where grade III indicates tumor aggressiveness and patient poor survival [13]. Similar results were also observed in other clinical breast cancer cohorts where miR-205-5p expression levels were measured by qRT-PCR [25, 26] and by *in situ* hybridization [27]. In addition to the miR-205-5p downregulation in high grade tissue biopsies, the presence of blood circulating miR-205-5p, indicating an overall high expression correlated with tumor chemo sensitivity and higher rates of disease free survival [28].

In all cases, miR-205-5p downregulation was linked to a worse prognosis. These findings correlate to our both observations in 21T cell lines and breast tumors highlighting the need of including miR-205-5p in more comprehensive clinical studies.

miR-205-5p has an established role in regulating epithelial to mesenchymal transition (EMT) and tumorigenesis [29–31]. When normally expressed, miR-205-5p can inhibit its targets such as ZEB1 and ZEB2 and consequently result in the expression of E-cadherin, keeping cell polarity and cell-cell junction integrity [32]. miR-205-5p validated targets, besides ZEB1 and ZEB2, include E2F1, HER3, VEGF-A, PTEN and NOTCH2 [6, 29, 33–35]. Our data support the notion that miR-205-5p downregulation contributes to breast cancer progression to metastasis. Derived from the same patient, 21T cells have a decreasing expression of miR-205-5p from non-tumoral H16N2 to metastatic 21MT1/2 consequently promoting higher proliferation behavior and tumor aggressiveness. In addition to the already known miR-205-5p targets we could by crossing mRNA and miRNA expression levels predict other genes involved in tumor progression that can contributing to the transition to a mesenchymal and more proliferative phenotype.

miRNAs can bind to their targets with a partial base pair complementarity. The level of expression of miRNA targets in a system is relevant for increasing the success rate in the target prediction [10]. By selecting just the mRNAs that were overexpressed in the transition *in situ* to metastatic cells, we take into consideration the specific cellular context allowing a more precise prediction.

Among our predicted targets, TGFB has already been experimentally validated in osteosarcoma cells [36]. However, other predicted targets such as SOCS3, PTPRN2, MMP3, PRG1 and BASP1 have not yet been experimentally validated but could be of particular interest on cancer research. SOCS3 draws special attention by having three miR-205-5p binding sites and for being able to promoting growth, metastasis and EMT in breast cancer [37, 38].

Our results show that cell migratory capacity inversely correlates with miR-205-5p expression, (Fig 4). We observed a lower migration rate in Boyden chamber after miR-205-5p transfection in 21MT1 and 21MT2 (S3). The opposite trend was also observed: higher migration rates were observed after miR-205-5p was silenced in non- invasive cell lines. Although not significant, it indicates that miR-205-5p contributes but it´s not the only factor responsible to 21T series invasiveness.

Altogether, our results reveal a shift on the miRNA expression during tumorigenesis in 21T cells and highlight miR-205-5p as a major player on breast tumor evolution from carcinoma *in situ* to metastasis.

## Conclusions

We analyzed differences in microRNA expression during breast cancer progression using the 21T series of cell lines. miR-205-5p appeared as the only deregulated microRNA in the transition carcinoma *in situ* to metastatic, showing a significant 5.4 times downregulation. This observation correlates to the cells invasive potential in Boyden chamber assay and was strengthened, with the *in vivo* downregulation of miR-205-5p in aggressive breast tumors. Our results show miR-205-5p as a tumor suppressor miRNA and contribute to understanding breast cancer progression to an invasive phenotype. Further studies will help to validate experimentally miR-205-5p target genes involved in this process.

## Supporting information

S1 File Fig A-D Differently expressed miRNA in 21T cell lines when compared to the non-tumoral H16N2 cell line.
(DOCX)

S2 File Table A: Characteristics of the breast formalin-fixed paraffin biopsies used in this study. Fig A: miR-205-5p expression levels in paraffin-embedded formalin fixed breast tissue samples.
(DOCX)

S1 Fig Changes in 21T cell lines status of invasiveness by switching miR-205-5p expression. (DOCX)

S1 Table miR-205-5p binding sites and estimated folding energy for the predicted targets. (DOCX)
