# Supplementary material for: The microRNA-205-5p is correlated to metastatic potential of 21T series: A breast cancer progression model
Source: PLoS One. 2017 Mar 27;12(3):e0173756. doi: 10.1371/journal.pone.0173756 (PMC5367783; doi:10.1371/journal.pone.0173756)
Supplement: S1 File — Fig A-D Differently expressed miRNA in 21T cell lines when compared to the non-tumoral H16N2 cell line. (DOCX) [file pone.0173756.s001.docx]

Figure A: Differently expressed miRNA in **21PT** when compared to **H16N2** cell line.


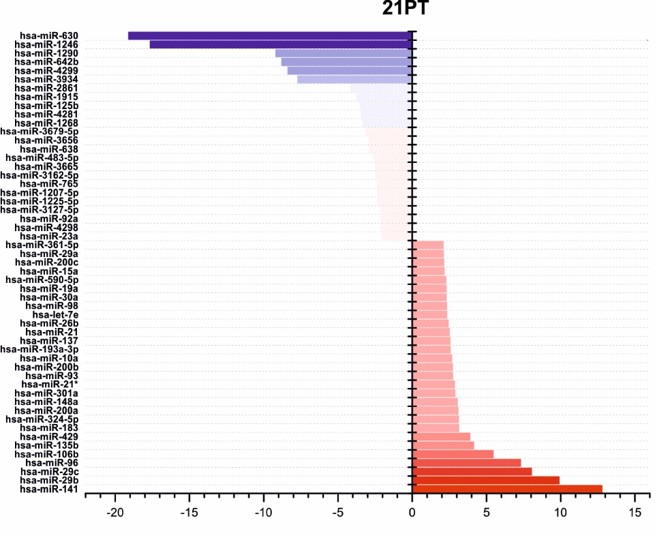


| ID | Fold Change | p-Value |
| --- | --- | --- |
| hsa-miR-630 | -19,096465 | 3,31E-04 |
| hsa-miR-1246 | -17,637083 | 2,99E-04 |
| hsa-miR-1290 | -9,1800787 | 1,37E-04 |
| hsa-miR-642b | -8,7849623 | 8,16E-05 |
| hsa-miR-4299 | -8,3648909 | 1,13E-03 |
| hsa-miR-3934 | -7,7001076 | 2,40E-04 |
| hsa-miR-2861 | -4,1511241 | 8,75E-04 |
| hsa-miR-1915 | -3,7198996 | 1,19E-03 |
| hsv2-miR-H10 | -3,5410104 | 2,27E-03 |
| hsa-miR-125b | -3,5017586 | 4,39E-04 |
| hsa-miR-4281 | -3,4208244 | 5,12E-04 |
| hsa-miR-1268 | -3,3130492 | 3,71E-04 |
| hsa-miR-3679-5p | -3,1095868 | 2,03E-04 |
| hsa-miR-3656 | -2,8985922 | 3,31E-04 |
| hsa-miR-638 | -2,8526286 | 1,34E-03 |
| hsa-miR-483-5p | -2,56878 | 2,40E-04 |
| hsa-miR-3665 | -2,4878059 | 1,10E-02 |
| hsa-miR-3162-5p | -2,4691395 | 8,10E-04 |
| hsa-miR-765 | -2,355339 | 3,62E-04 |
| hsa-miR-1207-5p | -2,3224921 | 5,83E-04 |
| hsa-miR-1225-5p | -2,3075727 | 7,62E-04 |
| hsa-miR-3127-5p | -2,1476942 | 1,65E-04 |
| hsa-miR-92a | -2,1237487 | 3,28E-03 |
| hsa-miR-4298 | -2,1121052 | 2,03E-04 |
| hsa-miR-23a | -2,0102504 | 1,10E-02 |
| hsa-miR-361-5p | 2,07379394 | 1,11E-03 |
| hsa-miR-29a | 2,09661366 | 2,40E-04 |
| hsa-miR-200c | 2,11365264 | 8,16E-03 |
| hsa-miR-15a | 2,15026377 | 1,65E-04 |
| hsa-miR-590-5p | 2,25818034 | 3,20E-02 |
| hsa-miR-19a | 2,2802864 | 9,74E-03 |
| hsa-miR-30a | 2,28611714 | 1,10E-02 |
| hsa-miR-98 | 2,31688686 | 9,44E-04 |
| hsa-let-7e | 2,32738732 | 1,69E-02 |
| hsa-miR-26b | 2,42561766 | 5,73E-03 |
| hsa-miR-21 | 2,49029503 | 4,55E-02 |
| hsa-miR-137 | 2,54094738 | 8,75E-04 |
| hsa-miR-193a-3p | 2,56276121 | 1,65E-04 |
| hsa-miR-10a | 2,66301507 | 1,65E-04 |
| hsa-miR-200b | 2,71011134 | 1,67E-02 |
| hsa-miR-93 | 2,71910634 | 5,23E-04 |
| hsa-miR-21* | 2,84467679 | 7,67E-03 |
| hsa-miR-301a | 2,86901311 | 2,32E-02 |
| hsa-miR-148a | 3,0310306 | 8,91E-04 |
| hsa-miR-200a | 3,08434241 | 1,22E-02 |
| hsa-miR-324-5p | 3,11104104 | 1,87E-03 |
| hsa-miR-183 | 3,1294566 | 1,37E-04 |
| hsa-miR-429 | 3,88072685 | 1,41E-03 |
| hsa-miR-135b | 4,13189153 | 8,20E-04 |
| hsa-miR-106b | 5,45008477 | 5,71E-04 |
| hsa-miR-96 | 7,2926313 | 1,65E-04 |
| hsa-miR-29c | 8,0239375 | 6,65E-03 |
| hsa-miR-29b | 9,88691871 | 6,50E-04 |
| hsa-miR-141 | 12,7805409 | 1,16E-03 |

Figure B: Differently expressed miRNA in **21NT** when compared to **H16N2** cell line.


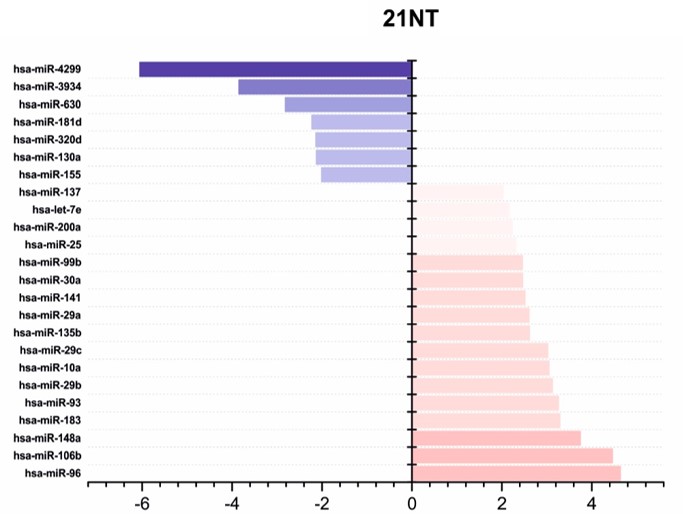


| ID | Fold Change | p-value |
| --- | --- | --- |
| hsa-miR-4299 | -6,05189991 | 2,14E-08 |
| hsa-miR-3934 | -3,84784043 | 1,04E-07 |
| hsa-miR-630 | -2,81557955 | 7,29E-09 |
| hsa-miR-181d | -2,22593301 | 3,45E-07 |
| hsa-miR-320d | -2,13840102 | 2,03E-08 |
| hsa-miR-130a | -2,12537564 | 2,03E-08 |
| hsa-miR-155 | -2,00913595 | 7,20E-08 |
| hsa-miR-137 | 2,02908535 | 6,80E-08 |
| hsa-let-7e | 2,16033233 | 7,24E-08 |
| hsa-miR-200a | 2,23479377 | 4,66E-07 |
| hsa-miR-25 | 2,31275523 | 1,10E-08 |
| hsa-miR-99b | 2,45695969 | 2,03E-08 |
| hsa-miR-30a | 2,46042523 | 2,03E-08 |
| hsa-miR-141 | 2,5130883 | 1,96E-08 |
| hsa-miR-29a | 2,60437515 | 7,29E-09 |
| hsa-miR-135b | 2,61686403 | 3,04E-08 |
| hsa-miR-29c | 3,0210972 | 2,03E-08 |
| hsa-miR-10a | 3,04976213 | 3,50E-09 |
| hsa-miR-29b | 3,12378129 | 3,42E-09 |
| hsa-miR-93 | 3,25799982 | 9,24E-09 |
| hsa-miR-183 | 3,290443 | 3,42E-09 |
| hsa-miR-148a | 3,74552348 | 3,81E-09 |
| hsa-miR-106b | 4,45844355 | 3,50E-09 |
| hsa-miR-96 | 4,63475082 | 3,42E-09 |

Figure C: Differently expressed miRNA in **21MT1** when compared to **H16N2** cell line.


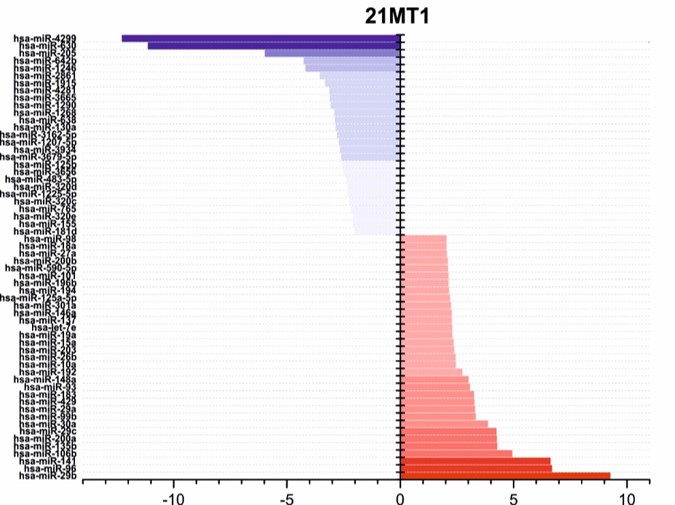


| ID | Fold Change | p-value |
| --- | --- | --- |
| hsa-miR-4299 | -12,2631 | 3,50E-10 |
| hsa-miR-630 | -11,1157 | 3,50E-10 |
| hsa-miR-205 | -5,95702 | 2,65E-09 |
| hsa-miR-642b | -4,24429 | 7,97E-10 |
| hsa-miR-1246 | -4,16882 | 3,09E-07 |
| hsa-miR-2861 | -3,52914 | 6,19E-10 |
| hsa-miR-1915 | -3,29937 | 8,46E-10 |
| hsa-miR-4281 | -3,10809 | 2,65E-09 |
| hsa-miR-3665 | -3,09026 | 2,18E-09 |
| hsa-miR-1290 | -3,0526 | 2,67E-07 |
| hsa-miR-1268 | -2,89104 | 2,08E-08 |
| hsa-miR-638 | -2,86446 | 3,10E-09 |
| hsa-miR-130a | -2,83316 | 1,58E-09 |
| hsa-miR-3162-5p | -2,76828 | 2,36E-09 |
| hsa-miR-1207-5p | -2,69068 | 3,60E-09 |
| hsa-miR-3934 | -2,64136 | 2,91E-07 |
| hsa-miR-3679-5p | -2,59014 | 2,65E-09 |
| hsa-miR-125b | -2,54738 | 1,55E-08 |
| hsa-miR-3656 | -2,49617 | 4,10E-08 |
| hsa-miR-483-5p | -2,36717 | 1,54E-08 |
| hsa-miR-320d | -2,31057 | 1,52E-08 |
| hsa-miR-1225-5p | -2,28982 | 1,18E-08 |
| hsa-miR-320c | -2,22435 | 2,24E-08 |
| hsa-miR-765 | -2,18228 | 2,76E-08 |
| hsa-miR-320e | -2,07447 | 2,92E-08 |
| hsa-miR-155 | -2,06611 | 1,37E-08 |
| hsa-miR-181d | -2,0105 | 9,92E-08 |
| hsa-miR-98 | 2,012698 | 3,34E-08 |
| hsa-miR-18a | 2,015224 | 2,08E-08 |
| hsa-miR-27a | 2,034992 | 4,31E-07 |
| hsa-miR-200b | 2,071429 | 2,33E-08 |
| hsa-miR-590-5p | 2,083447 | 1,13E-08 |
| hsa-miR-101 | 2,107646 | 5,77E-08 |
| hsa-miR-196b | 2,11048 | 2,33E-08 |
| hsa-miR-194 | 2,134801 | 1,15E-08 |
| hsa-miR-125a-5p | 2,168807 | 2,03E-08 |
| hsa-miR-301a | 2,21789 | 9,00E-09 |
| hsa-miR-146a | 2,249925 | 2,21E-08 |
| hsa-miR-137 | 2,252338 | 2,47E-08 |
| hsa-let-7e | 2,271636 | 6,53E-07 |
| hsa-miR-19a | 2,276478 | 5,08E-09 |
| hsa-miR-15a | 2,329875 | 5,09E-09 |
| hsa-miR-203 | 2,354109 | 1,96E-08 |
| hsa-miR-26b | 2,415259 | 7,77E-09 |
| hsa-miR-10a | 2,427143 | 5,69E-09 |
| hsa-miR-192 | 2,72133 | 9,41E-09 |
| hsa-miR-148a | 2,987291 | 3,60E-09 |
| hsa-miR-93 | 3,047865 | 4,30E-08 |
| hsa-miR-183 | 3,2314 | 1,02E-09 |
| hsa-miR-429 | 3,245654 | 1,00E-09 |
| hsa-miR-29a | 3,277029 | 1,58E-07 |
| hsa-miR-99b | 3,308683 | 2,36E-09 |
| hsa-miR-30a | 3,847199 | 5,44E-10 |
| hsa-miR-29c | 4,220459 | 3,60E-09 |
| hsa-miR-200a | 4,236243 | 1,00E-09 |
| hsa-miR-135b | 4,257226 | 2,67E-09 |
| hsa-miR-106b | 4,919436 | 6,19E-10 |
| hsa-miR-141 | 6,608673 | 3,50E-10 |
| hsa-miR-96 | 6,676471 | 3,50E-10 |
| hsa-miR-29b | 9,250347 | 1,02E-09 |

Figure D: Differently expressed miRNA in **21MT2** when compared to **H16N2** cell line.


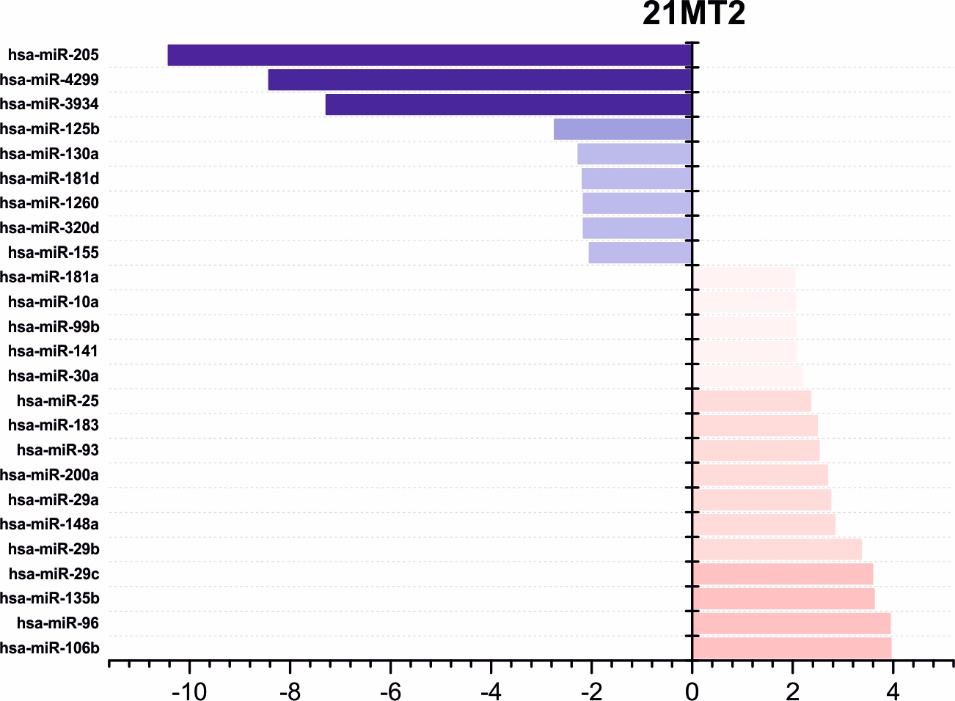


| ID | Fold Change | p-value |
| --- | --- | --- |
| hsa-miR-205 | -10,4205 | 1,27E-06 |
| hsa-miR-4299 | -8,42023 | 2,57E-06 |
| hsa-miR-3934 | -7,28101 | 2,54E-06 |
| hsa-miR-125b | -2,73694 | 4,57E-06 |
| hsa-miR-130a | -2,26547 | 1,53E-05 |
| hsa-miR-181d | -2,18375 | 6,38E-05 |
| hsa-miR-1260 | -2,16373 | 0,000255 |
| hsa-miR-320d | -2,16117 | 2,73E-06 |
| hsa-miR-155 | -2,04355 | 4,57E-06 |
| hsa-miR-181a | 2,037511 | 0,000505 |
| hsa-miR-10a | 2,050068 | 3,65E-06 |
| hsa-miR-99b | 2,054853 | 7,76E-05 |
| hsa-miR-141 | 2,070399 | 0,000115 |
| hsa-miR-30a | 2,187741 | 3,47E-05 |
| hsa-miR-25 | 2,355808 | 4,57E-06 |
| hsa-miR-183 | 2,486267 | 3,28E-06 |
| hsa-miR-93 | 2,521514 | 1,25E-05 |
| hsa-miR-200a | 2,691236 | 2,26E-05 |
| hsa-miR-29a | 2,750162 | 1,27E-06 |
| hsa-miR-148a | 2,83194 | 7,07E-06 |
| hsa-miR-29b | 3,360318 | 1,78E-05 |
| hsa-miR-29c | 3,586331 | 2,12E-06 |
| hsa-miR-135b | 3,618285 | 4,57E-06 |
| hsa-miR-96 | 3,93897 | 1,27E-06 |
| hsa-miR-106b | 3,95252 | 1,27E-06 |
