# Supplementary material for: The microRNA-205-5p is correlated to metastatic potential of 21T series: A breast cancer progression model
Source: PLoS One. 2017 Mar 27;12(3):e0173756. doi: 10.1371/journal.pone.0173756 (PMC5367783; doi:10.1371/journal.pone.0173756)
Supplement: S2 File — Table A: Characteristics of the breast formalin-fixed paraffin biopsies used in this study. Fig A: miR-205-5p expression levels in paraffin-embedded formalin fixed breast tissue samples. (DOCX) [file pone.0173756.s002.docx]

Table A: Characteristics of the breast formalin-fixed paraffin biopsies used in this study

| Tumor ID | Histological classification | Elston Grade | Tumor size | Lymph node metastasis |
| --- | --- | --- | --- | --- |
| T2 | Invasive ductal carcinoma | 1 | 2,0X1,2 cm | No data |
| T19 | Mucinous | 1 | ND | No data |
| T39 | Invasive ductal carcinoma | 1 | 0,6X0,5X0,4 cm | No data |
| T15 | Invasive ductal carcinoma | 2 | 1,5X1,2X1,0 cm | No data |
| T1 | Invasive ductal carcinoma | 2 | 2,7X1,7 cm | 1out of 16 lymph nodes |
| T18 | Invasive ductal carcinoma | 3 | 2,0X2,0X2,0 cm | 5 out of 18 lymph nodes |
| T40 | Invasive ductal carcinoma | 3 | 1,7X1,5 cm | 5 out of 11 lymph nodes |
| T72 | Invasive ductal carcinoma | 3 | 3,0X3,0X3,0 cm | absent |
| T36 | Invasive ductal carcinoma | 3 | 0,7 cm | No data |
| T26 | Invasive ductal carcinoma | 3 | ND | No data |
| T21 | Invasive ductal carcinoma | 3 | 0,9X0,7 cm | absent |
| N16/1 | Normal tissue |  |  |  |
| N16/2 | Normal tissue |  |  |  |
| N21 | Normal tissue |  |  |  |
| N26 | Normal tissue |  |  |  |
| N/5651.08.6 | Normal tissue |  |  |  |
| N/929.06.7 | Normal tissue |  |  |  |


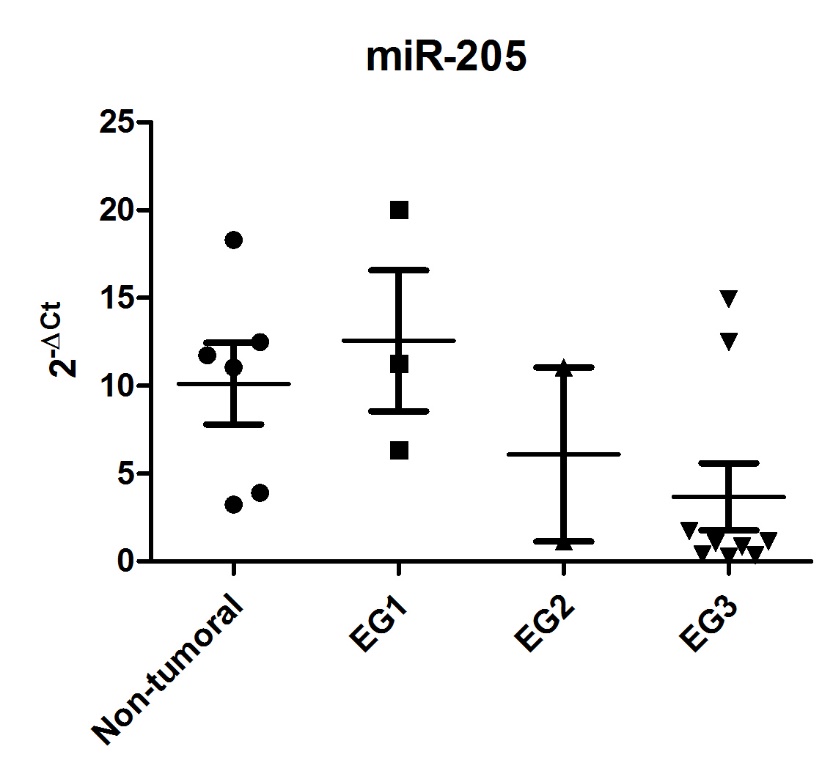


**Fig A: miR-205-5p expression levels in paraffin-embedded formalin fixed breast tissue samples.** miR-205-5p expression was accessed by qPCR as described in the material and methods. Graph display the distribution of expression level values without normalization to the non-tumoral sample. Expression levels were normalized to the reference gene b actin (ΔCT) and the expression level was obtained by 2^-ΔCT^
