# Supplementary material for: The microRNA-205-5p is correlated to metastatic potential of 21T series: A breast cancer progression model
Source: PLoS One. 2017 Mar 27;12(3):e0173756. doi: 10.1371/journal.pone.0173756 (PMC5367783; doi:10.1371/journal.pone.0173756)
Supplement: S1 Fig — (DOCX) [file pone.0173756.s003.docx]

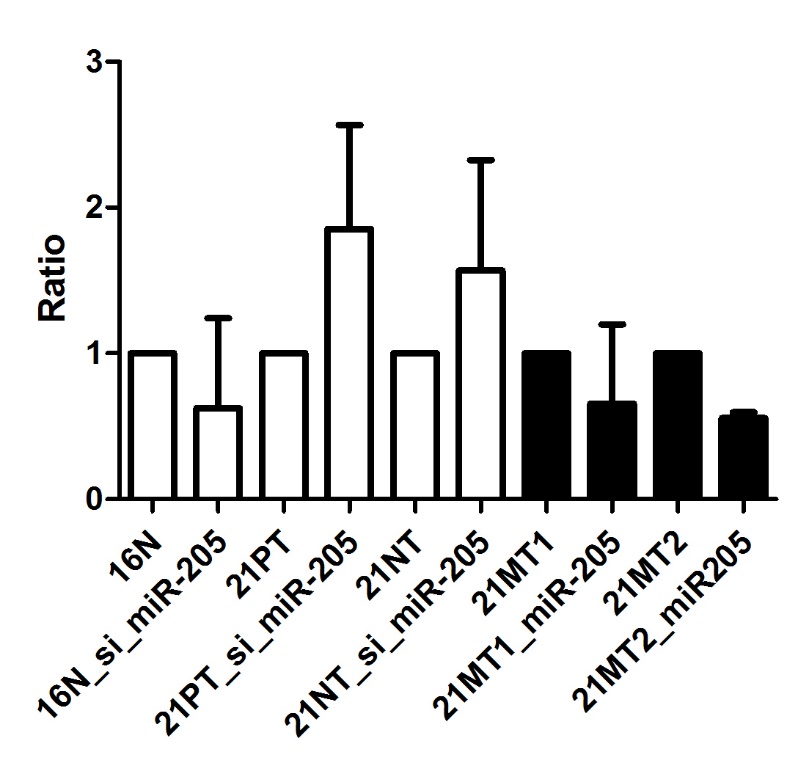


**S3 Fig: Changes in 21T cell lines status of invasiveness by switching miR-205-5p expression.**  21T cells migration capacity through an 8.0µm pore size polycarbonate membrane coated with Matrigel matrix. The non-invasive cell lines 16N, 21PT and 21NT were transfected with miR-205-5p silencer (si_miR-205-5p) and the metastatic cell lines 21MT1 and 21MT2 were transfected with miR-205-5p precursor (miR-205-5p). The number of migrating cells were assessed 22h after incubation at 37^o^C, 5% CO_2._ Results are displayed as a percentage ratio between transfected cells and their respective non-treated control.
