# Supplementary material for: The microRNA-205-5p is correlated to metastatic potential of 21T series: A breast cancer progression model
Source: PLoS One. 2017 Mar 27;12(3):e0173756. doi: 10.1371/journal.pone.0173756 (PMC5367783; doi:10.1371/journal.pone.0173756)
Supplement: S1 Table — (DOCX) [file pone.0173756.s004.docx]

S4 Table: miR-205-5p binding sites and estimated folding energy for the predicted targets

| **Gene symbol** | **Postion** | **Folding energy (Kcal/mol)** | **Heteroduplex** | **p-value** |
| --- | --- | --- | --- | --- |
| SOCS3 | 1635 | -25 | CTGGCTCCGG-GGA--GAAGGG | 0.043 |
|  |  |  | \|:\|\|\|\|\|\| \|\|\| \|\|\|\|\|: |  |
|  |  |  | GTCTGAGGCCACCTTACTTCCT |  |
|  |  |  |  |  |
| SOCS3 | 1650 | -19.3 | AGGGCTTGGGGTGACCTGAAGGG | 0.043 |
|  |  |  | :\|:\|\|: \|\| \|\| \|\|\|\|\|\|: |  |
|  |  |  | GTCTGAGGCCACCT-TACTTCCT |  |
|  |  |  |  |  |
| SOCS3 | 2230 | -15.9 | CTCCCTCCTGCCTGGG-TGGGGGA | 0.00547 |
|  |  |  | \|\|\|\| \| \|\|\|: \|\|::\|\|\| |  |
|  |  |  | GTCTGAGGCC--ACCTTACTTCCT |  |
|  |  |  |  |  |
| TGFB1 | 2072 | -18.4 | CAGACCTC--AGGAAAGAGGGG | 0.00528 |
|  |  |  | \|\|\|\|\| \| \|\|\|\| \|\|:\|\|: |  |
|  |  |  | GTCTGAGGCCACCTTACTTCCT |  |
|  |  |  |  |  |
| TGFB1 | 2355 | -16.1 | TGTGCATGTGG-GGGAGGAGGGA | 0.0282 |
|  |  |  | :\| \| :\|\| \|\|:\| \|\|:\|\|\| |  |
|  |  |  | GTCTG-AGGCCACCTTACTTCCT |  |
|  |  |  |  |  |
| PTPRN2 | 2481 | -14.8 | ACCACTCCCGGGTCCTGCTGAAGGC | 0.0102 |
|  |  |  | \|\|\|\|\| \|\| \|\|\|\|\|\| |  |
|  |  |  | GTCTGAGG-CC--ACCTTACTTCCT |  |
|  |  |  |  |  |
| MMP3 | 345 | -19.1 | CAGAAGTTCC-TTGGATTGGAGGT | 0.0055 |
|  |  |  | \|\|\|\| \|\|\| \|\|\|\| \|\|:\|\|\| |  |
|  |  |  | GTCT--GAGGCCACCTTACTTCCT |  |
|  |  |  |  |  |
| PRG1 | 170 | -12.92 | CAGTTTTCC-TCTCCTTGAAGGG | 0.054 |
|  |  |  | \|\|\| :\|\|\| \|\|\|\|\|\|: |  |
|  |  |  | GTC-TGAGGCCACCTTACTTCCT |  |
|  |  |  |  |  |
| BASP1 | 268 | -13 | CAAGCTCAGCAAGAAGAAGAAGGG | 0.00229 |
|  |  |  | \|\| \|\|\| \|\|\| \|\|\|\|\|: |  |
|  |  |  | GTCTGAGGCCACCTT--ACTTCCT |  |
|  |  |  |  |  |
| ENO2 | 2250 | -17.5 | CGGAGGCTGTGTG-CCTGGGGGA | 0.013 |
|  |  |  | \|:\|\| \|:\| \|\|\| \|\|::\|\|\| |  |
|  |  |  | GTCTGAGGC-CACCTTACTTCCT |  |
|  |  |  |  |  |
| TOSO | 1293 | -17.7 | CAGCCTGCCGCCATGATGGAGGA | 0.0525 |
|  |  |  | \|\|\| \|\| \|\|\| :\|\|\|:\|\|\|\| |  |
|  |  |  | GTCTGA-GGCCACCTTACTTCCT |  |
